# Seroepidemiology of Dengue Virus in Mayotte, Indian Ocean, 2006

**DOI:** 10.1371/journal.pone.0014141

**Published:** 2010-11-30

**Authors:** Daouda Sissoko, Khaled Ezzedine, Claude Giry, Amrat Moendandzé, Tinne Lernout, Eric D'Ortenzio, François Pettinelli, Denis Malvy

**Affiliations:** 1 Université Victor Segalen Bordeaux 2, Centre René Labusquière, Bordeaux, France; 2 Centre Hospitalier Universitaire, Service de Dermatologie, Hôpital Saint André, Bordeaux, France; 3 Centre Hospitalier de Mayotte, Laboratoire de Biologie, Mamoudzou, Mayotte, France; 4 Conseil Général de Mayotte, Direction de la Santé et de la PMI, Conseil Général BP 101, Mamoudzou, Mayotte, France; 5 Cellule de l'Institut de Veille Sanitaire en région Océan Indien, Mayotte, France; 6 Cellule de l'Institut de Veille Sanitaire en région Océan Indien, La Réunion, France; 7 Centre Hospitalier Universitaire, Service de Médecine Interne et de Maladies Tropicales, Hôpital Saint André, Bordeaux, France; St. Petersburg Pasteur Institute, Russian Federation

## Abstract

**Background:**

Although Dengue virus (DENV) circulation had been documented in neighbouring South-western Indian Ocean Islands, its presence in Mayotte is poorly characterised. To address this issue, we aimed to assess the seroprevalence of dengue IgG antibodies (DENV-IgG Ab) among the population and to investigate potential associations with individual and household characteristics.

**Methods/Principal Findings:**

In November–December 2006 we conducted a cross-sectional serologic survey in Mayotte among 1,154 inhabitants aged ≥2 years by using a multistage cluster random sampling method. The overall prevalence of DENV-specific IgG antibodies (ELISA) was 22.73% (95% CI, 18.16–27.31). The age-specific seroprevalence increased with age (*χ^2^* for trend = 11.86, P<0.0006), and was linked with previous known outbreaks in this region. In multivariate analysis, older age, being born in the Comoros and living in a household with a low socioeconomic index were positively associated with DENV IgG antibody positivity.

**Conclusions:**

These findings document substantial prior exposure of the population of Mayotte to DENV and highlight the risk of severe illness due to the possibility of sequential DENV infections. Further investigations characterizing current DENV circulation patterns and associated serotypes are needed.

## Introduction

Dengue is caused by one of four distinct dengue virus serotypes (DENV-1–4) belonging to the family of Flaviviridae, genus *Flavivrus*. Dengue infection is transmitted to humans through the bite of infected mosquitoes of the genus *Aedes*, commonly *Aedes aegypti* and *Aedes albopictus* [1]. The clinical picture of the illness may range from asymptomatic, non-specific acute febrile illness (dengue fever) to a fatal illness with hemorrhagic fever and circulatory shock known as dengue haemorrhagic fever/dengue shock syndrome.

During the last decades, dengue has evolved rapidly as viruses have spread worldwide expanding from Southeast Asia to the Caribbean and Latin American. Moreover, it is expected to disseminate further in receptive regions where competent vectors are present [2]. This has led to a change in the status of numerous countries, which have evolved from non-endemic (no serotype present) to hyperendemic (continuous co-circulation of multiple virus serotypes), as well as to an increase in the frequency of severe disease forms [3]. Currently, dengue is a pandemic with worrying public health implications throughout the tropics and subtropics. Indeed, DENV is considered as the most important re-emerging virus, directly threatening 2.5 billion people living in tropical/sub-tropical areas and resulting in around 50–100 million cases of dengue fever and 250 000 cases of dengue haemorrhagic fever, together with a mortality rate of 25 000 yearly [4].

Serological evidence of dengue infection has been reported in different countries or islands of the Eastern African costal region [5], [6], [7], [8], [9]. DENV-2 was the first serotype formally identified in the Southwest Indian Ocean Islands during the large outbreak that affected the Seychelles in 1976–1977 and Reunion Island in 1977–1978 with an estimated attack rate of respectively 60% and 35% [5], [10]. Subsequently, circulation of DENV-3 subtype III between the East-Africa coastal region, Southeast Asia and Latin American has been well characterised [11]. In addition, DENV-1 circulation was reported during the outbreaks of dengue both in the Comoros in 1993 (estimated attack rate of nearly 30%) [9] and Reunion in 2004 [12]. However, due to the lack of sustainable surveillance systems, available data are sparse in most of the countries of this region either during minor epidemics or during inter-epidemic periods. This is particularly noteworthy in Mayotte, where the only documentation of DENV circulation in the territory dates back to 1943 [13]. Since then, DENV circulation has been documented in neighbouring countries or islands which have frequent trade and population contact with Mayotte [5], [9], [10]. These islands share similar conditions that favour the spread of DENV, presence of dengue-competent vectors, comparable environmental and climatic conditions, lack of effective mosquito control, rapid population growth, uncontrolled urbanisation and inadequate water supply and waste management systems [14], [15], [16], [17].

Recently, the dramatic appearance of persistent regional and international outbreaks of Chikungunya virus (CHIKV) fever in 2005–2007 highlighted the vulnerability of the region to arthropod-borne viruses and their capacity for rapid expansion across the region and beyond [18], [19], [20]. These outbreaks have drawn attention to the need for better understanding of the local epidemiology of arboviruses in general and of DENV in particular. In order to provide reference data on previous DENV circulation in Mayotte, we have performed a serological survey with the goals of estimating the seroprevalence of DENV IgG-antibodies among individuals aged ≥2 years and to identify potential sociodemographic and environmental determinants of seropositivity.

## Materials and Methods

### Setting, design and population

**Figure 1:**
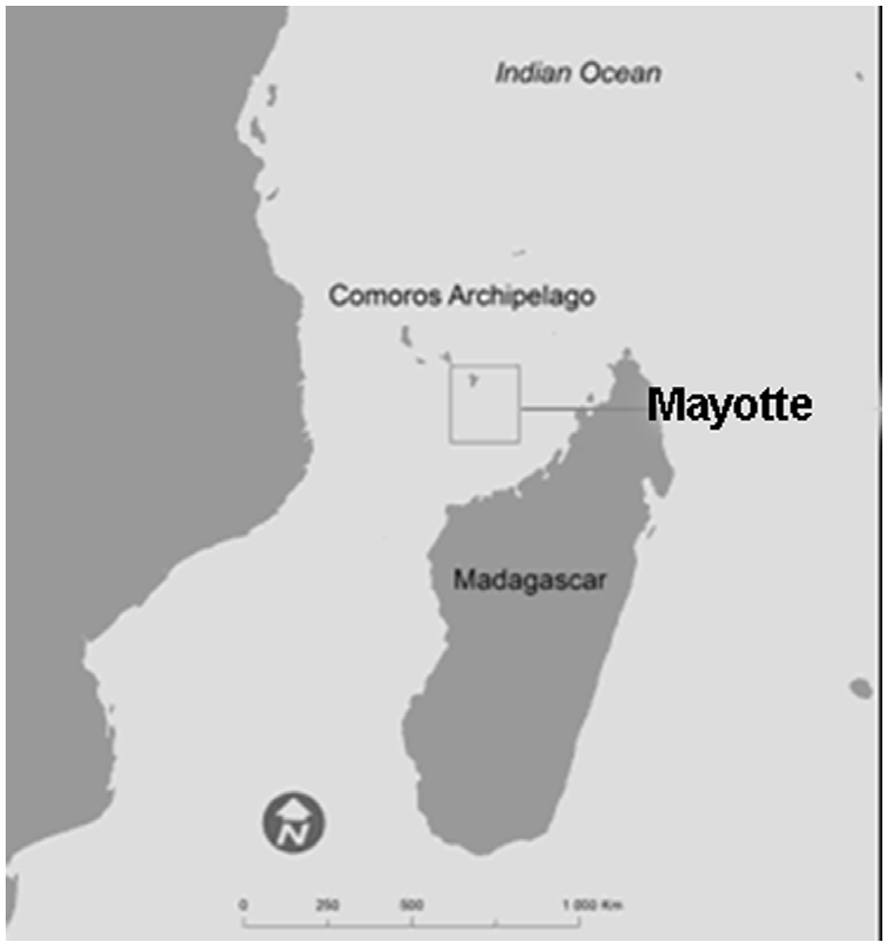
South-West Indian Ocean map with Mayotte.

The study was conducted in Mayotte, an insular French-administered territory located in the Comoros archipelago between the Eastern African coast and Madagascar (Figure 1). With regard to the 2002 census, the estimated population in 2006 was 175 000 with a density of 468 inhabitants per square km. The population originates primarily from Africa. Flow of immigrants from the neighbouring Comoro Islands accounted for approximately 35% of Mayotte's population. The study setting has been described previously [21]. The survey was carried out in conjunction with a CHIKV-related population-based serologic survey in November and December 2006, targeting the island population aged ≥2 years.

The survey used a multistage cluster sampling technique. In a first step, 40 of the 400 enumeration areas- EAs (clusters) provided by the 2002 census blocks were sampled randomly with probability proportionate to size, the size being the number of household in the cluster. Secondly, a systematic walk from a random starting point was performed to select 7 to 12 households (defined as groups of persons sleeping or eating together) within each EA. Finally, in each selected household, all adults (aged ≥15 years) and one individual aged 2–14 years (identified by using the next birthday selection method) were invited to participate in the survey [22]. Consequently, individuals belonging to the 2–14 year age group were under-represented in our sample compared to the general population, since 43% of total population of Mayotte is aged 0–14 years. To improve the level of participation, household visits included weekends and holidays. Moreover, when eligible members of the house were absent at the time of the initial visit, surveyors made up to two additional visits.

### Data collection

Participation in the survey was proposed on a voluntary basis. A pre-coded and pre-tested structured questionnaire was administered face-to-face at the house by trained surveyors in the local language (Shimaori). Sociodemographic data were collected relating to age, gender, educational level, country of origin and length of residence in Mayotte (for foreigners). Data were also collected on the housing environment, relating to the type of house construction (Makeshift, adobe and stone, concrete) the number of household residents, sanitary conditions and household facilities such as electricity and drinking water source. Data on the housing environment and on asset-ownership were collected with the collaboration of a reference person for the household.

A six-point household asset index was calculated as a proxy for socio-economic status for adults. This index was constructed on the basis of availability of electricity (1 point), a flush toilet within the household (1 point), piped water as source of drinking water (1 point), possession of a television set (1 point), radio (1 point), and a refrigerator (1 point). The number of items were summed for each household and the household assigned to one of two grades, namely low economic status (total score<median threshold) and medium to high socioeconomic status (total score ≥median threshold).

#### Laboratory methods

A venous blood sample was obtained from each participant. Immediately after puncture, blood samples were stored at 4–8°C and forwarded the same day to the laboratory of the Mayotte Territorial Hospital, which was responsible for all serologic testing. All serum samples were tested for specific IgG DENV antibodies as a marker of past infection using the Focus ELISA kit (Dengue Fever Virus IgG Focus Diagnostics, Cypress CA, USA). A commercial assay for the detection of IgG antibodies to dengue viruses was used (Focus Diagnostics, Cypress, CA, US). The test is an indirect IgG assay using a 96-well microtiter plate coated with equal proportions of inactivated, purified DENV types 1–4 (Type 1: TH-Sman; Type 2: TH-36; Type 3:H87; and Type 4: H241). Briefly, patient sera and controls were diluted 1∶101 in the kit diluent, and 0.1 ml was added to wells. Negative and positive controls were included, as was a kit-supplied calibrator/cutoff control. After incubation for 1 h at 37°C and washing three times with phosphate-buffered saline (PBS), peroxidase-labeled rabbit anti-human IgG conjugate was added to each well for 30 min. After the conjugation step and the final wash series with PBS, tetramethylbenzidine (TMB) was added, and the final reaction product was measured in a spectrophotometer at a wavelength of 450 nm. An index value was obtained for both control and patient samples by dividing the absorbance value of the patients and controls by the absorbance value of the calibrator (cutoff control). The optical density (OD) of the specimen well was compared with the OD of the well containing a calibration cut-off (CO) serum sample provided with the kit. Those samples with OD: CO ratio ≥1 were recorded as positive. The IgG ELISA assay used presented a sensitivity of 100% and a specificity of 88.3% [23].

### Statistical analysis

Data were double entered and corrected for data entry errors using EPIDATA® software version 3.0 (Epidata Association, Odense, Denmark). All analyses were performed with STATA® software version 10.0 (Stata Corporation, College Station, USA).

In the interest of representativity as regard to the reference population, each participant was weighted by post stratification according to age, gender, geographic location and household type in order to account for unequal probabilities of selection resulting from sampling cluster design of the study. For this purpose, sampling weights were prepared and provided by the Regional Bureau of the French National Institute for Statistics and Economic Studies using the 2006 population estimation on the basis of the 2002 census.

Crude and adjusted prevalence rates are presented by sociodemographic and household variables. Overall and subgroup-specific DENV seropositivity prevalence rates were estimated and differences within sub-groups were compared using binomial survey-adjusted Wald chi-square tests. P values<0.05 were considered statistically significant.

In a next step, explanatory bivariate analyses using population-adjusted weights to account for the survey design were performed to assess potential associations between DENV seropositivity (outcome of interest) and sociodemographic and housing variables. Crude odds ratios (OR) and their 95% confidence interval (95% CI) were estimated to explore the strength of the association between DENV seropositivity and the variable of interest. This analysis was only performed in the adult subgroup (≥15 years). Indeed, educational level and length of residence in Mayotte were not considered applicable for risk factor analysis for individuals aged 2–14 years.

Next, the association between variables identified in the bivariate analysis and the outcome of interest were explored by multivariable logistic regression analysis in order to control for confounders. For this purpose, stepwise logistic regression models including sociodemographic and household variables were performed. All variables significantly associated with seropositivity at a probability threshold of ≤0.25 in the bivariate analyses were entered into the initial multivariate models. For this purpose, 36 individuals were excluded due to missing data for at least one of the variables of interest, leaving a total of 852 observations. A backward stepwise procedure was used to select variables retained in each of the final models [24]. Furthermore, in order to retain a final model, a two-by-two interaction terms were checked for variables that remained statistically significant in the pre-final model. The strength of the association between selected variables and DENV seropositivity was estimated by adjusted odds ratios (AOR) with their 95% CI, all AOR excluding 1.0 being considered as significant. In order to reflect adequately association with outcome, confidence intervals for AOR estimates were calculated on the basis of a log transformation with the standard errors computed by the delta method that took into account both clustering of individual observations within households and non-independence of the variables at household and region levels [25].

### Ethical considerations

Publicity and information about the survey was provided through the media, field visits to the local population, and contact with local and national authorities. During visits, surveyors introduced themselves to selected households and explained the objectives and all procedures to the household members concerned. A written consent form was first obtained from adults or guardians of those individuals aged <18 years prior to the inclusion, unless they were illiterate, in which case signed consent was sought from an appropriate family member chosen by the participant. As the survey was combined with the determination of CHIKV seroprevalence, it is important to note that we solicited the agreement for testing several arboviruses including DENV. This study was reviewed and approved by the ethics committee of the Bordeaux University Hospital Centre, France, in compliance with all French regulations on protection of human subjects. A numbered code was attributed to all participants to track blood samples. All data were handled confidentially and anonymously.

## Results

### Characteristics of participants and prevalence of serum DENV antibodies

Forty villages (clusters) were sampled and 508 inhabited household visited. Of these, 418 households (82.3%) agreed to participate in the study. The mean household size was 5.7±2.4 individuals [range: 1 to 13]. The selection procedure allowed the recruitment of a total sample of 1156 individuals. Two participants in the 2–14 years age group provided insufficient serum to undergo serological evaluation and were thus excluded from the analysis.

Comparison of the sociodemographic characteristics of the study sample to the reference demonstrated an adequate agreement. However, the study sample was under-represented in men compared to women (43·2% vs. 50·2% in 2006 population projection) as well as over-represented in individuals belonging to the 15–24-years age-group (25.5% vs. 20.2% in the 2006 population projection). These minor differences were accounted for in the analyses of seroprevalence and risk factors by allocating a weight to each participant.

The overall weighted seroprevalence of DENV antibodies in Mayotte for individuals aged ≥2 years was 22.7% (95% CI, 18.2–27.3) (Table 1). The age-specific weighted prevalence increased with age (χ2 for trend = 11.86, P<0.0006). The peak age-specific prevalence was observed in individuals of the 25–54 year age group while those of the 2–14 age group presented the lowest prevalence rate. When the analysis was restricted to individuals born in Mayotte, the prevalence rate was highest in individuals aged over 35 years (1.6% in the 2–14 age group, 4.6% in the 14–24age group, 6.2% in the 25–34 age group, 15.7% in the 35–44age-group, 22.2% in the 45–54 age group and 23.6% in individuals over 55 years of age). Of the 302 children (age group 2–14), eight had serum DENV antibodies (weighted prevalence: 2.2%); Of these eight children, three were less than 10-years old and born in Mayotte. No difference in the seroprevalence of DENV antibodies was observed according to gender (Table 1). The highest seroprevalence percentages were observed in the Northern and the North-Eastern part of the island (Table 2).

**Table 1:** Weighted prevalence of serum IgG DENV antibodies according to demographic variables in participants aged ≥2 years, Mayotte 2006.

Characteristic	Tested individuals (N)	Weighted prevalence (%)	95% Confidence interval
N (number of participants)	1154	22.7	18.2–27.3
Gender			
Male	499	21.8	16.5–27.2
Female	655	23.6	18.7–28.6
Age group, years			
02–14	302	2.2	0.7–3.7
15–24	294	19.1	12.9–25.6
25–34	193	34.0	24.9–43.1
35–44	169	35.8	23.0–48.7
45–54	107	38.8	23.7–53.8
≥55	89	28.4	17.4–39.4
Birthplace*			
Mayotte	446	10.6	6.8–14.5
Comoros	349	51.8	45.6–58.1
Other	60	17.3	1.7–32.9
Duration of residence, years †			
<10	211	52.1	41.2–62.9
10–14	84	49.7	32.8–66.6
Over 15	108	36.9	25.9–47.9
Years of schooling*			
0–6	679	31.3	24.8–37.7
>6	173	16.6	10.4–22.8

*Persons≥15 years.

†For those adults (≥15 years) born outside of Mayotte.

**Table 2:** Weighted prevalence of serum anti-DENV IgG antibodies according to household variables in individuals aged ≥2 years, Mayotte, 2006.

Variable	Tested individuals (N)	Weighted prevalence, %	95% Confidence interval
**House construction type**			
Makeshift	101	36.30	27.23–45.37
Adobe and stone	345	27.03	20.25–33.81
Concrete	708	18.89	13.76–24.02
**Household size (number of inhabitants)**			
1–2	101	29.94	23.55–36.33
3–4	276	21.36	13.63–29.09
≥5	777	18.40	13.36–23.45
**Region of residence**			
Northeast	417	24.94	17.33–32.56
North	255	25.97	14.91–37.02
Midwest	303	17.66	9.59–25.73
South	179	19.91	14.45–23.37
**Socioeconomic Asset index**			
Below the median	288	32.90	27.38–38.42
At or above the median	866	19.68	14.83–24.53

### Association between sociodemographic and household variables and DENV seropositivity

In the bivariate analysis, DENV seropositivity was significantly associated with a number of demographic and environmental variables. For example, seropositivity was positively associated with being born in another Comoro island (OR: 8.60; 95% CI: 5.56–13.27), whereas it was negatively associated with time at school ≥6 years (OR = 0.42, 95% CI: 0.26–0.66) and a longer duration of residence in Mayotte. Individuals who had resided in Mayotte for ≥15 years were protected from past infection as compared to those who had resided for less than 5 years (OR = 0.18, 95% CI: 0.10–0.34). Moreover, living in a household below the median asset index threshold or with a larger number of household members (3–4 individuals) was significantly associated with DENV seropositivity (Table 3).

**Table 3:** Unadjusted odds ratios for occurrence of serum anti-DENV IgG antibodies according to demographic and environmental variables in individuals aged ≥15 years, Mayotte, 2006.

	Crude odds ratio	95% Confidence interval	P
**Individual variables**			
Gender			
Male	0.94	0.70–1.27	0.71
Female	1.00		
Age, years			
15–24	1.00		
25–34	2.15	1.27–3.66	0.005
35–44	2.33	1.40–3.90	0.002
45–54	2.65	1.22–5.74	0.015
≥55	1.66	0.85–3.24	0.136
Birthplace			
Mayotte	1.00		
Comoros	8.60	5.56–13.27	<10^−3^
Other	1.76	0.55–5.53	0.33
Duration of residence, years			
<5	1.00		
5–14	1.08	0.55–2.11	0.24
Over 15	0.18	0.10–0.34	<10^−3^
Years of schooling			
0–6	1.00		
≥6	0.42	0.26–0.66	<10^−3^
**Household variables**			
Socioeconomic Asset index			
Below the median	1.00		
At or above the median	0.47	0.32–0.70	<10^−3^
Household size (number of inhabitants)			
1–2	1.00		
3–4	3.34	1.62–6.88	0.002
≥5	1.44	0.77–2.67	0.238
House construction type			
Makeshift	1.00		
Adobe and stone	0.57	0.29–1.11	0.10
Concrete	0.35	0.20–0.52	<10^−3^

In the multivariate logistic regression model, only two demographic variables remained significantly associated with DENV seropositivity, namely increasing age and being born in another Comoros island (Table 4). Concerning household-related variables, living in a household of three to four members, as well as living in makeshift house and a socioeconomic status index below the median threshold were positively associated with DENV seropositivity (Table 4).

**Table 4:** Adjusted odds ratios for occurrence of anti-DENV IgG antibodies according to demographic and environmental variables in individuals aged ≥15 years, Mayotte, 2006.

	Adjusted odds ratio	95% Confidence interval	P-value
**Individual variables**			
Age, years			
15–24	1.00		0.0006
25–34	2.13	1.094.18	
35–44	2.79	1.45–5.35	
45–54	4.76	1.85–12.22	
≥55	3.08	1.27–7.45	
Birthplace			
Mayotte	1.00		
Comoros	10.29	6.70–15.83	<10^−3^
Other	1.62	0.54–4.80	0.37
Years of schooling			
0–6	1.00		
≥6	0.74	0.46–1.22	0.23
**Household variables**			
Socioeconomic Asset index			
Below the median	1.00		
At or above the median	0.61	0.38–0.99	0.048
Household size (number of inhabitants)			
1–2	1.00		
3–4	4.46	1.61–7.45	0.002
≥5	2.10	1.40–3.16	0.15
House construction type			
Makeshift	1		
Adobe and stone	0.66	0.35–1.26	0.20
Concrete	0.49	0.29–0.86	0.014

## Discussion

This is the first large-scale survey of the prevalence of dengue infection and its potential determinants that has been conducted in Mayotte. The overall weighted prevalence of IgG DENV antibodies was 22.7%, ranging from 2.2% in children to almost 40% for individuals aged 45–54 years. This increasing age-related prevalence of IgG DENV is consistent with previous studies conducted in other endemic settings [26], [27]. We observed that previous DENV infection in adults was independently associated with increasing age, low socio-economic status and household size. In contrast, no difference in seroprevalence was found for gender. Previous reports of potential gender differences in dengue infections have been inconsistent, with some finding a higher prevalence in men, others a higher prevalence in women, and others no gender difference, in line with our findings [28], [29], [30]. This inconsistency may be related to variations in gender-specific exposure according to local cultural practices.

The study identified a striking association of IgG DENV prevalence with certain sociodemographic variables. Comorian natives immigrating to Mayotte aged 15 years or over were around ten-fold more affected than individuals of the same age born in Mayotte. This finding may be, at least in part, related to the outbreak of dengue that occurred in the neighbouring Comoros islands in 1993 [9]. This epidemic hardly affected Mayotte, which may explain the association observed.

Moreover, the age distribution of IgG DENV in the native population of Mayotte reinforces this hypothesis. The peak of IgG DENV prevalence in this group is observed in individuals aged 35 years or more, suggesting a link with the major dengue outbreak that affected the South-Western Indian Ocean islands, in 1976–1978, which was first reported in the Seychelles (1976–1977) [5] and subsequently in Reunion (1977–1978) [6]. Although this outbreak was well documented in these two countries, DENV circulation in Mayotte was not reported at the time. However, it can be speculated that the 1976–1978-DENV outbreak had in fact probably spread to Mayotte as well, as did the recent persistent regional outbreak of CHIKV infection, first reported in Reunion. Another important finding is the evidence of past infection among younger individuals, particularly those native to Mayotte and born after the Comoros outbreak of 1993. This may suggest potentially that low level transmission of DENV in Mayotte has continued during the last decade. This is the first suggestion of inter-epidemic transmission that has been described in Mayotte.

With respect to household variables, a higher risk for previous DENV infection was found in households with a socio-economic threshold below the median. In addition, there was a positive association between household size and DENV seroprevalence, which was restricted to households with three to four residents. This particular finding may be related to the structure of the questionnaire that assessed the number of inhabitants per household rather than the density of inhabitants per room. The latter variable may be more pertinent for evaluating potential associations between DENV circulation and housing.

Our study has a number of limitations that should be considered. Firstly, it was a cross sectional study relying on past exposure, rather than a prospective incidence study. Secondly, specific documentation of DENV serotypes that firmly establish the circulation of different viral variants was not performed due to logistic and financial constraints. On the other hand, this study was conducted on a representative sample of the target population, which is one of the strengths of the study. In particular, it was weighted with respect to age, sex, and housing conditions relevant to the local population, and also took into account local migratory flux.

The advantage of using commercial ELISA to perform a high throughput testing for DENV serology screening is well documented [31], [32]. ELISA testing allows detection of all serotypes (DEN-1–4) and has a similar performance as the hemagglutination test, considered to be the gold standard for indirect serologic diagnosis of DENV infection [33]. Besides, ELISA testing for DENV may also detect cross-reactive antibodies to other flaviviruses, such as West Nile virus, whose circulation has been reported in Madagascar [10]. However, it should be noted for the limitation of this assumption that yellow fever has never been reported and that immunization against the disease is neither recommended by French Health authorities nor by the US CDC. This is true for local inhabitants as well as for travellers to the island. Moreover, no epidemic of West Nile virus has yet been notified either in Mayotte or in Reunion Island.

This is the first cross-sectional study to demonstrate the circulation of DENV in Mayotte using methods that discriminate between different age groups and between different origins of subjects. Hence DENV infection should be considered in the differential diagnosis of influenza-like illnesses in routine clinical practice in Mayotte. Moreover, routine laboratory surveillance should be enhanced to improve the accuracy of detection of viral circulation trends, including the identification of the specific DENV serotypes involved. In parallel, community-specific habits, customs or behaviours that increase the risk of mosquito-borne infections should be identified to facilitate implementation of prevention campaigns by health services and to educate the general population.

A lot has been learnt from previous major outbreaks of arboviral infections in the South-Western Indian Ocean region, notably during the recent chikungunya virus epidemic. Continuous efforts should be put into developing regional cooperation to set up early warning systems and to ensure prompt responsiveness to counter rapidly-spreading vector-borne viruses as due to the presence in Mayotte of two competent vectors *Aedes aegypti* and *Ae. Albopictus* [*17*].

Finally, from a global health perspective, this region at the crossroads of Asia and Africa should be more thoroughly monitored for dengue virus circulation, due to the increase in intercontinental exchanges and legal and illegal immigration.
